# Outbreak response capacity of the Global Outbreak Alert and Response Network across WHO’s South-East Asia and Western Pacific regions

**DOI:** 10.5365/wpsar.2024.15.5.1109

**Published:** 2024-08-12

**Authors:** Amy Elizabeth Parry, Sai Campbell, Stefan Thottunkal, Partha Pratim Mandal, Sharon Salmon

**Affiliations:** a Department of Applied Epidemiology, National Centre for Epidemiology and Population Health, College of Health and Medicine, The Australian National University, Canberra, Australia.; b World Health Organization Regional Office for South-East Asia, New Delhi, India.; c World Health Organization Regional Office for the Western Pacific, Manila, Philippines.; d Indo-Pacific Centre for Health Security, Department of Foreign Affairs and Trade, Canberra, Australia.; e UNSW Medicine, School of Public Health and Community Medicine, University of New South Wales, Sydney, New South Wales, Australia.

## Abstract

**Objective:**

The COVID-19 pandemic challenged the Global Outbreak Alert and Response Network’s (GOARN) mechanism used to rapidly deploy technical support for international responses and highlighted areas that require strengthened capacity within the Network. GOARN’s partners in the World Health Organization’s (WHO) South-East Asia and Western Pacific regions were engaged to explore their levels of preparedness, readiness and ability to respond to international public health emergencies.

**Methods:**

Consultative discussions were held and a survey was conducted with GOARN’s partners from the two WHO regions. Discussion topics included partners’ capacity to support and participate in a GOARN deployment, training, research and collaboration. Descriptive and content analyses were conducted.

**Results:**

Barriers to engaging in GOARN’s international outbreak response efforts included limited numbers of personnel trained to respond to outbreaks; institutional, financial and administrative hurdles; and limited collaboration opportunities. Partners identified innovative solutions that could strengthen their engagement with deployment, such as financial subsidies, mentorship for less experienced staff, and the ability to provide remote support.

**Discussion:**

GOARN plays an important role in enabling WHO to fulfil its international alert and response duties during disease outbreaks and humanitarian crises that have the potential to spark disease outbreaks. Yet without systematic improvement to strengthen national outbreak capacity and regional connectedness, support for international outbreak responses may remain limited. Thus, it is necessary to integrate novel approaches to support international deployments, as identified in this study.

The COVID-19 pandemic tested and stretched health systems and the health workforce. The pandemic presented unique challenges to international collaboration for emergency responses and emphasized the critical importance of partnerships and developing innovative solutions to address global health emergencies.

Established by the World Health Organization (WHO) in April 2000, the Global Outbreak Alert and Response Network (GOARN) comprises technical institutions and networks from around the world. GOARN can pool resources to coordinate response activities and facilitate information-sharing about emerging and ongoing public health events. When requested by WHO Member States, GOARN coordinates its partners’ technical capacities to respond to a public health emergency. (*1*)

WHO’s South-East Asia and Western Pacific regions are home to more than one quarter of the world’s population. Both regions are prone to natural disasters, disease outbreaks and health risks from climate change. (*1*) In September 2022, GOARN comprised 270 partners internationally, including 22 (8%) in the South-East Asia Region and 63 (23%) in the Western Pacific Region. (*2*)

The COVID-19 pandemic created an opportunity for GOARN’s partners to reflect on their institutional capacities for preparedness and response activities. This study aimed to explore the enablers and barriers to partners’ engagement with GOARN to strengthen international responses to and preparedness and readiness for global health emergencies after the COVID-19 pandemic. This opportunity also provided a platform for partners within the two regions to contribute to the drafting of the GOARN Strategy 2022–2026. (*3*)

## Methods

Small-group discussions were conducted with GOARN’s partners in the Western Pacific Region. Subsequently, partners from both regions responded to a survey designed to document their preparedness, readiness and capacity to respond to acute public health events.

### Group discussions

In May 2022, all 63 GOARN partners in the Western Pacific Region were invited via e-mail to attend a virtual discussion. Partners in the South-East Asia Region were not available for this component of the study. Invitations were extended to GOARN focal points, but they were transferrable to other personnel. More than one person from each institution could attend, if available. Participation in the group discussions was voluntary; all participants were verbally informed that notes would be taken during the discussions but no data about the individuals or institutions participating in the discussions would be collected (e.g. demographic data or role within the GOARN partner institution). Participants self-selected into one of two separate online, interactive sessions. Each session included standard questions, was facilitated by the same two staff from The Australian National University and lasted approximately 90 minutes.

To encourage interaction, participants were asked to answer questions by responding verbally or using the chat function on Zoom. Participants were also asked to respond to a series of short-answer questions using the Mentimeter online tool (Stockholm, Sweden). Topics explored in the discussions included partners’ activities undertaken in relation to preparedness and response, challenges and enablers to participating in GOARN, perceptions of the value of partnering with GOARN, and what support would be required to encourage partners to become more active. Participants were also consulted about the draft GOARN strategy document.

Discussion notes, as well as digital data from Zoom and Mentimeter, were categorized and descriptively analysed. Inductive content analysis was used to group common concerns and issues raised by participants. These identified concerns were then used to inform the development of the subsequent survey of GOARN’s partners.

### Survey

GOARN’s partners from both regions were invited to complete one self-administered online survey per institution using REDCap (Research Electronic Data Capture; REDCap Consortium, Vanderbilt University, Nashville, TN, USA). Survey questions covered four main themes: institutional preparedness and readiness, deployment, operational research and collaboration. Closed and open-ended questions were included. The survey link was distributed to 85 GOARN focal points in the two regions (Western Pacific, *n* = 63; South-East Asia, *n* = 22) via e-mail and was open during 5–25 July 2022. Consent was obtained online before access to the survey was provided. Survey data were analysed using Microsoft Excel (2016) and Stata 15 (StataCorp, College Station, TX, USA). Two researchers conducted qualitative inductive content analysis to identify patterns and groupings in the responses to the open-ended questions. (*4*)

### Dissemination

Study results were presented and discussed at a biregional online meeting of GOARN’s partners on 23 August 2022. A report was then finalized and distributed to all partners in these two regions. (*5*)

## Results

Sixty-six individuals from invited GOARN partner institutions participated in the two group discussions (group 1, *n* = 36; group 2, *n* = 30). Analysis of the group discussions identified concerns and challenges regarding partners’ ability to deploy, partners’ involvement in GOARN training, collaboration with GOARN and with other partners, and partners’ capacity to participate in operational research about emergency responses. Survey questions were developed to address these areas.

The overall survey response rate was 55% (47/85): 48% (30/63) of partners from the Western Pacific and 77% (17/22) of partners from the South-East Asia regions. Respondents were from 13/48 Member States within the two WHO regions (Western Pacific, 8/37; South-East Asia, 5/11). One duplicate partner survey was removed, and all remaining data were included in the analysis. The majority of respondents were from partners at universities and government organizations. All GOARN technical capacities were represented (**Table 1**).

**Table 1:** Technical characteristics of survey respondents at GOARN’s partner organizations in WHO’s South-East Asia and Western Pacific regions, 2022 (*n* = 47)

Technical area	No. (%)
**Infection prevention and control, protection of the health workforce**	**29 (62)**
**Surveillance and risk assessment**	**29 (62)**
**Operational research, implementation science, monitoring and evaluation**	**29 (62)**
**Laboratory services and diagnostics**	**27 (57)**
**Case management, clinical operations, therapeutics research**	**19 (40)**
**Case investigation, contact tracing**	**12 (26)**
**Implementation of GOARN projects or tools to improve data collection, harmonization and analysis during outbreaksa**	**11 (23)**
**Coordination and planning**	**10 (21)**
**Risk communication and community engagement, infodemic management**	**10 (21)**
**Vaccinations**	**9 (19)**
**Points of entry, international travel and transport, mass gatherings**	**8 (17)**
**Maintaining essential health services and systems**	**4 (9)**
**Operational support and logistics, supply chains**	**2 (4)**

GOARN: Global Outbreak Alert and Response Network.

^a^ GOARN’s projects or tools include Go.Data and integrated outbreak analytics.

### Theme 1: partner preparedness and readiness

Approximately one third (17/47, 36%) of respondents reported staff or member participation in any tier of the GOARN Capacity-Strengthening and Training Programme. In terms of training delivery, 74% (35/47) of respondents indicated they had not yet delivered a GOARN training, but they might in the future have the capacity and interest to deliver such training.

### Theme 2: deployment

Forty-five per cent (21/47) of respondents reported deploying personnel to an international response with GOARN since becoming a partner (**Table 2**). Forty per cent (19/47) stated that their institution was “deployment-ready” and able to deploy immediately if requested.

**Table 2:** Ability of GOARN’s partners to engage in deployment activities in WHO’s Western Pacific and South-East Asia regions, survey results, 2022 (*n* = 47)

Deployment	No. (%) of responses		
	Yes	No	Unsure
**By institution type**			
**Governmental organization**	**8 (53)**	**2 (13)**	**5 (33)**
**Ministry or Department of Health**	**1 (50)**	**0 (0)**	**1 (50)**
**Hospital**	**5 (45)**	**3 (27)**	**3 (27)**
**International organization**	**1 (50)**	**1 (50)**	**0 (0)**
**Professional network**	**0 (0)**	**2 (100)**	**0 (0)**
**Nongovernmental organization**	**3 (43)**	**0 (0)**	**4 (57)**
**Special programme**	**1 (100)**	**0 (0)**	**0 (0)**
**University**	**7 (44)**	**4 (25)**	**5 (31)**
**Research institute**	**6 (55)**	**4 (36)**	**1 (9)**
**By GOARN pillar**			
**Infection prevention and control, protection of the health workforce**	**15 (50)**	**4 (13)**	**11 (37)**
**Surveillance and risk assessment**	**16 (55)**	**2 (7)**	**11 (38)**
**Operational research, implementation science, monitoring and evaluation**	**11 (38)**	**4 (14)**	**14 (48)**
**Laboratory services and diagnostics**	**11 (41)**	**6 (22)**	**10 (37)**
**Case management, clinical operations, therapeutics research**	**10 (53)**	**4 (21)**	**5 (26)**
**Case investigation, contact tracing**	**8 (67)**	**0 (0)**	**4 (33)**
**Implementation of GOARN projects or tools to improve data collection, harmonization and ** ** analysis during an outbreak**	**9 (82)**	**0 (0)**	**2 (18)**
**Coordination and planning**	**5 (50)**	**0 (0)**	**5 (50)**
**Risk communication and community engagement, infodemic management**	**5 (50)**	**1 (10)**	**4 (40)**
**Vaccinations**	**5 (56)**	**2 (22)**	**2 (22)**
**Points of entry, international travel and transport, mass gatherings**	**4 (50)**	**0 (0)**	**4 (50)**
**Maintaining essential health services and systems**	**2 (50)**	**1 (25)**	**1 (25)**
**Operational support and logistics, supply chains**	**2 (100)**	**0 (0)**	**0 (0)**

GOARN: Global Outbreak Alert and Response Network; ND: no data.

Respondents expressed interest in exploring virtual deployment, with 31/47 (66%) reporting interest in engaging in this style of deployment if it was offered. Respondents also suggested that their capacity to support international deployments would increase if less experienced personnel could be paired with or shadow experienced experts (**Table 2**).

Results showed that to enable the deployment of staff, it would be important to ensure continuation of pay or salary (38%, 18/47), provide leave to respond (36%, 17/47) and backfill deployed employees (19%, 9/47) (**Table 3**). Other enabling strategies included being able to delegate work responsibilities and having procedures to facilitate non-personal leave.

**Table 3:** Strategies used by GOARN’s partners in WHO’s South-East Asia and Western Pacific regions to enable staff to deploy, 2022 (*n* = 47)

Strategy	No. (%)
**Continuation of pay**	**18 (38)**
**Provision of leave**	**17 (36)**
**Backfill employee**	**9 (19)**
**Other**	**8 (17)**
**None**	**14 (30)**

Respondents reported multiple barriers to deploying personnel through GOARN. Administrative barriers included financial, human resources and contractual challenges, as well as long institutional approval times for deployments. Broader barriers included family commitments, competing domestic response priorities, routine work commitments and the requested duration of deployment being too long.

### Theme 3: operational research

Operational research was identified as integral to improving the effectiveness of GOARN’s activities and its overall preparedness and response. Respondents identified a range of institutional capacities relating to operational research, with 40% (19/47) reporting that their institution was able to undertake rapid literature reviews and 28% (13/47) reporting they were able to assist with ethics reviews. Respondents noted that any research undertaken should be translated into action and that data-sharing between partners could be improved. Respondents expressed an interest in collaborating on operational research with other GOARN partners across the regions.

### Theme 4: collaboration

To strengthen partnerships across the regions, the study explored partners’ reasons for engaging with GOARN and their interest in and capacity for interpartner collaborations. The key motivations for participating were to engage in capacity-strengthening and training (75%, 35/47) and in networking among partners (72%, 34/47); to take part in international deployments for outbreak response (64%, 30/47) and operational research (64%, 30/47); and to support response preparedness (47%, 22/47). The most useful GOARN activities were trainings, deployments, networking and communication of outbreak information, including through webinars.

Eighty-one per cent (38/47) of respondents expressed an interest in collaborating with other GOARN partners. Regular opportunities for networking were perceived by many as key to increasing collaboration.

## Discussion

This study identified that GOARN’s partners in the South-East Asia and Western Pacific regions have richly diverse experiences and technical capacities across each of GOARN’s areas of work. The study identified partners’ interest in collaborating and their capacity to do so. Partners involved in this study shared innovative ideas around how to strengthen emergency responses.

Capacity-building was identified as a key priority for partners. Partners identified actions that could scale up and sustain emergency response training, as well as challenges to be considered and addressed. Traditionally, GOARN’s training has been conducted by the GOARN Operational Support Team; (*6*) this study highlighted the potential to expand the emergency-ready workforce through partner-delivered trainings. Greater partner involvement in delivering GOARN training could help scale up emergency response workforce preparedness, creating teams equipped with the necessary skills to respond to acute national, regional and international public health events.

The potential to take part in international deployments for emergency responses was identified as a core purpose for joining GOARN. Partners in the South-East Asia and Western Pacific regions acknowledged their intent to support international deployments; however, multiple barriers to fulfilling this intent were reported. Respondents identified innovative solutions to address these barriers, including developing support mechanisms, such as mentorships for less experienced members of the workforce during deployment. Mentorship during emergency response is possible and can improve the workforce’s capacity to respond. (*7*, *8*) Developing a mentorship programme that pairs experienced, previously deployed team members with those who are inexperienced may strengthen and increase participation, Network collaboration and capabilities.

Respondents from universities or linked to universities indicated a willingness to deploy. GOARN could benefit from exploring these opportunities to create closer collaborations with universities and identify individuals from these partners who are ready to respond. Partners who were not able to deploy immediately wanted to further explore how they could in the future. GOARN would benefit from identifying alternative, acceptable deployment modes that are family-friendly, such as remote deployments. Collaborating with partners to support them to deploy will strengthen these regions’ capacity to provide quality responses to public health emergency events.

The importance of operational research to ensure an improved, evidence-informed response has been underscored in the GOARN Strategy 2022–2026. (*3*) This study found that GOARN’s partners across the South-East Asia and Western Pacific regions have a range of institutional capacities in operational research, which are presently underutilized. Developing a collaborative operational research agenda for emergency preparedness and response, along with a regional community of practice, would support the discovery of new and stronger ways to navigate this space.

This study has helped GOARN gain a better understanding of the capacities of its partners, as well as their needs. To take advantage of the information gleaned from and momentum created by this study and to strengthen communication and collaboration with partners, GOARN could map its membership by areas of technical expertise, desired collaboration areas, and collaboration capacity to GOARN and to each of its partners. Partners have voiced their interest in developing stronger connections with GOARN and among partners. The Network would benefit from creating technical or location-based hubs to improve collaboration, connection and engagement among partners.

This project successfully engaged many of GOARN’s partners from WHO’s South-East Asia and Western Pacific regions; however, there were limitations. First, GOARN’s partners from the South-East Asia Region were not able to participate in the first round of the study, the group discussions. To counterbalance this, the authors consulted with WHO’s GOARN staff to ensure regional input. Second, as partners were asked to provide only one survey response per institution, some institutional memory or content may have been unintentionally omitted. Dissemination of the final deidentified report to partners has helped to address this limitation.

GOARN plays a critical role in enabling WHO to fulfil its international alert and response duties during disease outbreaks and humanitarian crises that have the potential to spark disease outbreaks. (*9*) Continued strengthening of GOARN’s operational capacity to respond will ensure its preparedness for future public health emergencies at the national and international levels.

## References

[1] Asia Pacific strategy for emerging diseases and public health emergencies (APSED III): advancing implementation of the International Health Regulations (2005): working together towards health security. Manila: WHO Regional Office for the Western Pacific; 2017. Available from: https://iris.who.int/handle/10665/259094, accessed 21 December 2023.

[2] Global Outbreak Alert and Response Network. About us: partner institutions. Geneva: World Health Organization; n.d. [Internet]. Available from: https://goarn.who.int/about/partners, accessed 28 August 2023.

[3] Global Outbreak Alert and Response Network (GOARN). strategy 2022–2026. Geneva: World Health Organization; 2023. Available from: https://iris.who.int/handle/10665/366066, accessed 28 August 2023.

[4] Zhang Y, Wildemuth BM. Qualitative analysis of content. In: Wildemuth BM, editor. Applications of social research methods to questions in information and library science. Westport (CT): Libraries Unlimited; 2009. pp. 308–19.

[5] Global Outbreak Alert and Response Network (GOARN) partners meeting on international outbreak response capacity in the Asia-Pacific Region 2022: meeting report. Manila: WHO Regional Office for the Western Pacific; 2022. Available from: https://iris.who.int/handle/10665/366656, accessed 28 August 2023.

[6] Christensen R, Fisher D, Salmon S, Drury P, Effler P. Training for outbreak response through the Global Outbreak Alert and Response Network. BMC Med. 2021 May 14;19(1):123. 10.1186/s12916-021-01996-5 33985496 PMC8118749

[7] Parry AE, Colquhoun S, Brownbill A, Lynch BM, Housen T. Navigating uncertainty: evaluation of a COVID-19 surge workforce support program, Australia 2020–2021. Glob Biosecurity. 2021;3:124. 10.31646/gbio.124

[8] Parry AE, Colquhoun SM, Field E, Kirk MD, Durrheim DN, Housen T. How can we better support the public health emergency response workforce during crises? West Pac Surveill Response. 2021 Nov 23;12(4):1–3. 10.5365/wpsar.2021.12.4.886 35572737 PMC8873918

[9] Mackenzie JS, Drury P, Arthur RR, Ryan MJ, Grein T, Slattery R, et al. The global outbreak alert and response network. Glob Public Health. 2014;9(9):1023–39. 10.1080/17441692.2014.951870 25186571 PMC4205922

